# Combination of physical activity and screen time on life satisfaction in adults: A cross-sectional survey

**DOI:** 10.3389/fpsyg.2022.962520

**Published:** 2022-10-04

**Authors:** Zhenhuai Chen, Jiangang Sun, Wei Zhuang

**Affiliations:** ^1^Faculty of Physical Education, China West Normal University, Nanchong, China; ^2^Faculty of Physical Education, West Anhui University, Lu’an, China; ^3^School of Physical Education, South China University of Technology, Guangzhou, China; ^4^School of Physical Education and Sport Training, Shanghai University of Sport, Shanghai, China

**Keywords:** physical activity, television viewing, psychological wellbeing, adults, population health

## Abstract

**Background:**

Sufficient physical activity (PA) and limited screen time (ST) have been shown to be positively associated with a variety of mental health outcomes. It has been known that PA and ST are independently associated with life satisfaction. Whereas, little is known about the association between combinations of PA and ST with life satisfaction in adults. This study aimed to explore the associations between PA and ST (in insolation or combination) and life satisfaction in adults.

**Methods:**

Data from the 2014 European Social Survey (ESS) round 7 consisting of 22 countries were analyzed in this study. In total, self-reported data from 40,185 adults were included in the final analysis. The self-administered method was used to collect demographic information, PA, ST, and life satisfaction. The prevalence of meeting PA guidelines (at least 150 min per week) and ST guidelines (no more than 3 h per day) was calculated according to Canadian 24-h Movement Guidelines for Adults.

**Results:**

Adults who were engaged in sufficient PA and limited ST were more likely to report a higher level of life satisfaction. Meeting PA or ST guidelines was more likely to report higher life satisfaction scores [odds ratio (OR) = 1.31, 95% CI: 1.16–1.47]. Compared with not meeting any guidelines, those who met both PA and ST guidelines had a higher OR (OR = 1.55, 95% CI: 1.37–1.76).

**Conclusion:**

This study found that participating PA while limiting ST concurrently was linked with better life satisfaction. Creating an active lifestyle is important to population’s well-being.

## Introduction

A judgment of life satisfaction can be made on the basis of a person’s extensive evaluation of life. This is the subjective evaluation of their personal experience. It is a self-report measure (Pavot and Diener, 2008), and people are required to score their overall life satisfaction from 0 to 10. However, the distribution of life satisfaction in the Organization for Economic Co-operation and Development (OECD) countries is uneven. Moreover, the overall life satisfaction of some countries, such as Colombia, Greece, South Korea, Portugal, and Turkey, is relatively low, and the average score is less than 6. Relatively, the scores in Denmark, Finland, Iceland, the Netherlands, and Switzerland could be 7.5 or even higher (OECD) and show that men’s life satisfaction increases with age, but the results for women are different. In early and late adulthood, the life satisfaction scores of the two sexes diverge. For both women and men in early and late middle age, the score is insignificant. The predictive variables’ intensity is varied in middle-aged people, especially for people in late middle age (Medley, 1980). Research indicates that life satisfaction is associated with psychological health results (Nes et al., 2013), which can forecast mortality (St John et al., 2013). Based on two recent reviews of healthy people, life satisfaction is related to mortality (Chida and Steptoe, 2008). Additionally, it is linked with other positive results, such as job performance, organizational commitment, and turnover intentions (Erdogan et al., 2012). Life satisfaction is associated with a series of impressive results. In a recent meta-analysis, life satisfaction was linked to a reduction in mortality, and wellbeing was associated with a reduction in cardiovascular mortality (Chida and Steptoe, 2008). Other benefits include lower levels of sleep complaints (Brand et al., 2010) and burnout syndrome (Zavaleta et al., 2017).

In 2009, physical inactivity was considered one of the major risk factors leading to non-communicable diseases, and millions of deaths that could have been prevented have been caused by it (World Health Organization, 2009). Nevertheless, about one-third of adults in the world do not perform enough exercise to keep healthy (Hallal et al., 2012). As reported by studies performed in Australia (Milligan et al., 2007), Canada (Bryan and Katzmarzyk, 2009), the United States of America (Piercy et al., 2018), and Europe (Gerovasili et al., 2015; Marques et al., 2015), more than 33% of adults spend only a little time on exercise in their daily life, even though physical activity (PA) benefits health (Powell and Pratt, 1996; Warburton and Bredin, 2019). A number of institutions, such as the World Health Organization (WHO), the U.S. Department of Health and Human Services, and the European Union, have recommended that healthy adults should spend 30 min or longer time performing aerobic activities of moderate intensity for at least 5 days a week if they wish to stay healthy (Andersen et al., 2008; Fulton and Kohl, 2008; World Health Organization, 2010). Moreover, sedentary behavior (SB) refers to behaviors that expend energy less than 1.5 metabolic equivalents, such as watching television, lying down, and sitting (Tremblay et al., 2017). Most published works that have conducted a survey on SB based on population have been conducted in developed countries, and there is only a little worldwide data on adults in this respect. Recently, there was an assessment based on accelerometer usage that was a part of research involving a large-scale demographical survey of representative respondents, and it showed that adults spend a median of 8.2 h every day on SB (ranging from 4.9 to 11.9) (Bauman et al., 2018). Adults spend 9 or 10 h on SB every day in America and Canada (Prince et al., 2020; Matthews et al., 2021). It seems that adults spend a long time on SB than previously expected, and most of it is during leisure time and work (Matthews et al., 2021). Previous studies also provided evidence that SB, such as watching TV, leads to hampered mental health (Huang et al., 2020). In 2018, the health-related recommendations on PA were updated by the WHO based on the latest findings in science, and SB was also discussed in this aspect (World Health Organization, 2018).

In the future, life satisfaction and PA will be changed (Heller et al., 2006; Conroy et al., 2011; Maher et al., 2013). People can restore their vitality, reduce fatigue, and increase pleasure by engaging in more PA every day (Puetz et al., 2006; Reed and Ones, 2006). When daily PA is increased, people are inclined to be more satisfied with their life as compared to days of a regular amount of PA (Maher and Conroy, 2017), such as during interpersonal communication. Previous results of research concerning the elderly and middle-aged people are identical in terms of the following: a normal level of PA is related to satisfaction *via* fitness and health-related adaptation that improve mental and physical health. However, based on surveys of respondents of the younger generation, the general level of PA does not have much relation to life satisfaction. However, there is scarce research on the relationship between life satisfaction and daily PA among the elderly and the middle-aged, but there is a possible relationship between daily PA and life satisfaction among all age groups (Maher and Conroy, 2017) because it has been found that PA has an immediate recovery effect on all adults. Moreover, according to recent evidence, life satisfaction might be lower among the elderly and younger generations due to declines and challenges in developmental stages (Arnett, 2000; Gerstorf et al., 2008, 2010). Moreover, life satisfaction is greater among middle-aged people, partly because they gradually turn their attention inward to themselves rather than others, such as their children (Lachman and Firth, 2004). Increased sedentary time may be associated with a decline in life satisfaction among those between 18 and 25 years old; however, at these ages, the levels of PA are decreasing (Troiano et al., 2008). It remains unknown whether there is any relationship between SB and life satisfaction or between physical inactivity and life satisfaction. Despite limited studies on the relationship between life satisfaction and SB in adults, it is possible that sedentary people—for example, those who watch TV—also show decreased life satisfaction on average (Frey et al., 2007; Depp et al., 2010).

To our knowledge, no study has analyzed the relationship between PA and screen time (ST) on life satisfaction in adults using European Social Survey (ESS) data. This study aimed to test the associations between PA and ST (in insolation or combination) and life satisfaction in adults.

## Materials and methods

### European Social Survey (2014)

The ESS is an academically driven, cross-border survey that has been conducted throughout Europe since 2001. Every 2 years, a new cross-sectional sample is determined, and participants are interviewed in person. The ESS measures attitudes, beliefs, and behavior patterns in a diverse group of people. In the 2014 ESS (round 7), the survey covered 22 countries. The 2014 ESS selected a representative sample of countries consisting of residents aged 15 and older; self-report data were collected from 40,185 adults, except for the homeless and the sheltered. The 2014 ESS was funded by members, observers, and guests of the ESS European Research Infrastructure Alliance (ESS ERIC), which represents governments. Participating countries directly finance ESS ERIC’s central coordination costs, as well as fieldwork and national coordination costs in their own countries. The 2014 ESS included strict random probability sampling, a minimum target response rate of 70%, and strict translation protocols. During the 1-h face-to-face interview, participants were asked questions on various core themes repeated in previous rounds of the ESS, along with two modules developed for round 7 on social inequality in health (ESS, 2014). More information on the ESS, such as questionnaires and data collection, can be found on the ESS website, i.e., https://ess-search.nsd.no/en/study/ccd56840-e949-4320-945a-927c49e1dc4f.

### Physical activity and screen time

Information about PA was obtained through a single item that assessed the frequency of spending at least 30 min on walking very fast, exercising, or engaging in other PA in the past week (On how many of the last 7 days did you walk quickly, do sports, or other PA for 30 min or longer?). Options ranged from “0” to “7 days.” Previous studies have shown that the item is reliable (Wanner et al., 2014).

Participants’ average amount of time spent watching TV per day (how much time, in total, and do you spend watching TV on an average weekday) was also assessed (Santos et al., 2022). Based on the previous study (Keadle et al., 2015), options ranged from “no time at all” to “more than 3 h,” with an interval of 30 min.

According to Canadian 24-h Movement Guidelines for Adults aged 18–64 years and adults aged 65 and older, adults need to participate in PA for at least 150 min per week (PA guideline) and no more than 3 h of ST (ST guidelines) (Ross et al., 2020). This study used question options of 5–7 days to calculate the attainment of PA guidelines.

### Life satisfaction

Life satisfaction was assessed by the item “how satisfied are you with your whole life?” The answers were indicated on a scale ranging from 0 “extremely dissatisfied” to 10 “extremely satisfied.” Previous research suggested that such an item could be a robust indicator and reliably estimate life satisfaction (Cheung and Lucas, 2014).

### Covariates

Sex (male/female), age (years), years of education, body mass index (BMI), marital status, number of household members, and household income level were included as covariates. Regarding marital status, respondents were asked whether they lived with their husband/wife/partner and their legal status. In terms of the number of household members, respondents were asked whether they had children and how many family members they lived with regularly. Household income was determined in tenths.

### Statistical analysis

All statistical analysis was performed using SPSS version 23.0. Results were weighted based on the complex sampling survey design, and the weighted percentage of the sample was reported. Descriptive statistics were used to report sample characteristics (covariates), independent variables, and outcome variables. Logistic regression models were used to explore the associations of PA, ST, and their combinations with life satisfaction, adjusting for sex, age, years of education, BMI, marital status, number of household members, and household income level. The statistical significance level was set to *p* < 0.05, as previously illustrated.

## Results

### Demographic characteristics

The characteristics of participants’ demographics and socio-economic statuses are shown in Table 1. The average age of the participants was 47.5 years, with a balanced gender proportion of 51.5% women. The mean BMI in study participants was 25.6, while the mean year of education was 12.9. Each participant had approximately three household members, while more than half (52.1%) of the participants were legally married. In terms of socioeconomic status, participants were evenly distributed between the 1st decile and the 10th decile, with percentages ranging from the lowest of 8.7% in the 1st decile to the highest of 10.7% in the 4th and 7th deciles.

**TABLE 1:** Sample characteristics of this study.

		Estimate	95%CI	
**Age (mean)**		47.5	47.1	47.8
**Body mass index (mean)**		25.6	25.5	25.7
**Years of education (mean)**		12.9	12.8	13.0
**Number of household member (mean)**		2.9	2.9	2.9
**Sex%**				
	Male	48.5%	47.7%	49.3%
	Female	51.5%	50.7%	52.3%
**Marital status%**				
	Legally married	52.1%	51.3%	52.9%
	In a legally registered civil union	1.3%	1.1%	1.5%
	Legally separated	0.4%	0.3%	0.5%
	Legally divorced/civil union dissolved	8.0%	7.6%	8.4%
	Widowed/civil partner died	6.8%	6.4%	7.2%
	None of these (never married or in legally registered civil union)	31.4%	30.7%	32.2%
**Household income level%**				
	1st decile	8.7%	8.2%	9.3%
	2nd decile	10.0%	9.4%	10.5%
	3rd decile	10.4%	9.9%	10.9%
	4th decile	10.7%	10.2%	11.3%
	5th decile	10.3%	9.8%	10.8%
	6th decile	10.5%	9.9%	11.0%
	7th decile	10.7%	10.1%	11.2%
	8th decile	10.4%	9.8%	10.9%
	9th decile	8.9%	8.4%	9.5%
	10th decile	9.5%	8.9%	10.1%

### Prevalence of life satisfaction, physical activity, and screen time

As shown in Table 2, more than half (52.5%) of the participants had have high levels of life satisfaction, while 39.9% of the participants have medium levels of life satisfaction. In terms of PA days, 25.6% of the participants responded that they did not participate in PA even once a week, while approximately a quarter of the participants reported that they performed PA 7 days a week. A total of 4.8 and 6.4% of the participants, respectively, reported that they did not watch or watched less than 0.5 h of television per day, while the highest percentage of participants who watched more than 3 h of television per day was 18.0%. According to the comparison of PA and ST guidelines, most of the participants (67.0%) did not meet the PA guidelines, whereas the majority of the participants met the ST guidelines (82.0%). Near three-fifths (67.1%) of the participants met either of the guidelines, 12.9% met neither, and 27.9% met both, respectively.

**TABLE 2:** Results for independents and outcome.

		Estimate	95%CI	
**Life satisfaction**				
	Low	7.6%	7.2%	8.1%
	Medium	39.9%	39.1%	40.7%
	High	52.5%	51.6%	53.4%
**Physical activity days**				
	0 days	25.6%	24.9%	26.4%
	1 day	10.3%	9.8%	10.8%
	2 days	11.4%	10.9%	11.9%
	3 days	11.9%	11.4%	12.5%
	4 days	7.8%	7.4%	8.3%
	5 days	7.8%	7.4%	8.3%
	6 days	3.4%	3.1%	3.8%
	7 days	21.7%	21.0%	22.4%
**Television viewing time**				
	No time at all	4.8%	4.5%	5.2%
	Less than 0.5 h	6.4%	6.0%	6.8%
	0.5–1 h	14.0%	13.5%	14.6%
	More than 1 h, up to 1.5 h	14.5%	13.9%	15.0%
	More than 1.5 h, up to 2 h	16.5%	15.9%	17.2%
	More than 2 h, up to 2.5 h	13.7%	13.1%	14.3%
	More than 2.5 h, up to 3 h	12.1%	11.5%	12.6%
	More than 3 h	18.0%	17.4%	18.7%
**Physical activity guidelines**				
	Not meet	67.0%	66.2%	67.8%
	Meet	33.0%	32.2%	33.8%
**Screen time guidelines**				
	Not meet	18.0%	17.4%	18.7%
	Meet	82.0%	81.3%	82.6%
**Combination of physical activity and screen time**				
	Meet neither	12.9%	12.3%	13.5%
	Meet either	59.2%	58.4%	60.1%
	Meet both	27.9%	27.1%	28.6%

### Association between physical activity, screen time, and life satisfaction

**FIGURE 1:**
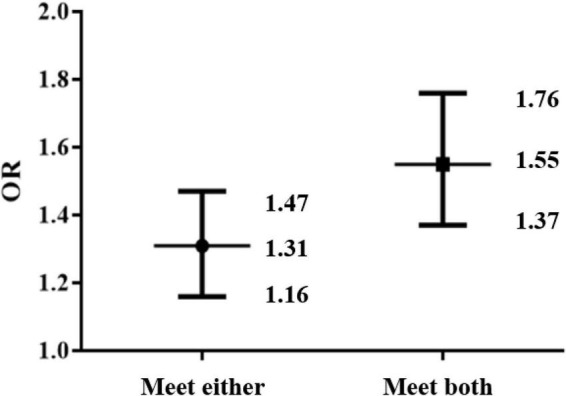
The associations between physical activity and screen time guidelines (in insolation or combination) and life satisfaction.

The results of multivariable regression are presented in Table 3. In terms of PA days, we observed that participation in PA greater than or equal to 1 day per week was a positive factor for higher life satisfaction. According to ST, watching television for more than 0 h per day was a negative factor for higher life satisfaction. In Figure 1, we can see that meeting either guidelines [odds ratio (OR) = 1.31 (1.16, 1.47)] and meeting both guidelines [OR = 1.55 (1.37, 1.76)] were positive factors for higher life satisfaction.

**TABLE 3:** Results for the associations between physical activity, televising viewing time, and life satisfaction (higher).

		OR	95%CI	
**Physical activity days**				
	0 days	Reference		
	1 day	1.22	1.06	1.40
	2 days	1.33	1.18	1.51
	3 days	1.45	1.28	1.65
	4 days	1.49	1.28	1.73
	5 days	1.43	1.23	1.67
	6 days	1.35	1.08	1.68
	7 days	1.51	1.36	1.69
**Television viewing time**				
	No time at all	Reference		
	Less than 0.5 h	0.92	0.73	1.15
	0.5–1 h	0.82	0.68	0.98
	More than 1 h, up to 1.5 h	0.85	0.71	1.03
	More than 1.5 h, up to 2 h	0.84	0.69	1.02
	More than 2 h, up to 2.5 h	0.80	0.66	0.97
	More than 2.5 h, up to 3 h	0.76	0.62	0.93
	More than 3 h	0.67	0.55	0.81

Adjusted for sex, age, years of education, body mass index (BMI), marital status, number of household members, and household income level.

## Discussion

Participating in more PA and higher ST was positively and negatively associated with life satisfaction, respectively. Compared with meeting neither of the guidelines, participants meeting either of the guidelines in isolation were more likely to report higher life satisfaction scores. Moreover, meeting both PA and ST guidelines might be related to higher life satisfaction scores.

There is a positive correlation between PA and increased life satisfaction, and this was consistent with previous research. Prior studies clarified that daily PA might have a top-down or bottom-up impact on life satisfaction. From a top-down perspective, more physically active people should report overall higher life satisfaction (Maher et al., 2013). This study showed that overall PA might improve physical health, while daily PA might reduce anxiety, or improving self-esteem could reinforce life satisfaction. Prospective research on the elderly indicated that regular participation in sports activities was indirectly associated with improved life satisfaction (Elavsky and McAuley, 2005; Elavsky et al., 2005; McAuley et al., 2008). Studies showed that PA could indirectly improve life satisfaction by affecting emotional health, self-efficacy, and mental health (Elavsky and McAuley, 2005; Elavsky et al., 2005; McAuley et al., 2008). Moreover, from a bottom-up perspective, being more or less physically active on a given day than usual should affect a person’s life satisfaction for that day. The level and intensity of PA decrease throughout adulthood (Troiano et al., 2008), and these changes might help to explain the decline in life satisfaction during this development period; however, it is unclear whether the association between PA and life satisfaction found in the elderly is applicable to adults. However, the current research results indicate that publicity or minor changes in daily might be a way to offset the decline in life satisfaction in adulthood (Maher et al., 2013).

Previous studies implied that prolonged screening might also lead to poor health (Biswas et al., 2015; Zhai et al., 2015; Wang et al., 2019; Mougharbel and Goldfield, 2020). Studies indicated that ST was related to depressive symptoms in adults (Madhav et al., 2017). There have been different findings on the association between SB and life satisfaction when using subjective and objective measurements. Research showed that there was no correlation between self-reported SB and life satisfaction in the elderly (Maher and Conroy, 2017), revealing the effects on perceived time use rather than the actual sitting time. Understanding the nature of sedentary activity might have an important impact on the relationship between SB and life satisfaction (Maher and Conroy, 2017). Research revealed that different changes in types and ST on weekdays and weekends affect the intensity of the relationship between screen-based SB and depressive symptoms. A survey indicated that 66 and 88% of adults spent more than 2 h on the screen on weekdays and weekends, respectively (Schoeppe et al., 2016). Beyond that, studies indicated that an increased risk of depression was linked to the long-time use of mobile phones for at least 2 h on weekdays but at least 5 h on weekends (Liu et al., 2019). The extent to which adults perceive certain sedentary activities as beneficial is an important finding of future research because it is relevant to identifying which sedentary activities increase or decrease life satisfaction.

The results of this study suggested that meeting both PA and ST guidelines was possibly linked to cumulatively higher life satisfaction during adulthood. This research also demonstrated that decreased PA and increased ST were associated with higher levels of negative mental health and lower positive mental health. Almost all participants with greater ST reported higher levels of negative mental health and lower levels of positive mental health as compared to participants with less ST. ST is often defined as psychologically passive SB, which can be explained by the correlation between ST and mental health (Meyer et al., 2020). There is a significant correlation between life satisfaction and mental health, implying that life satisfaction may be a psychological predictor (Bao et al., 2013). Moreover, studies realized that those who met PA guidelines but were more sedentary, as well as those with insufficient PA and less sedentary time, were more inclined to be obese than those with adequate PA and less sedentary time (Sugiyama et al., 2008). In other words, participants in the PA-deficient/high ST category were significantly more likely to be overweight relative to the active PA/low ST category (Liao et al., 2011). Studies manifested that both perceived and actual weight exerted an impact on people’s level of life satisfaction, and it has also been shown that BMI and perceived weight were linked to a higher probability of having a low level of life satisfaction (Herman et al., 2013).

This study adds to the evidence about the associations between PA, ST, and life satisfaction. Moreover, due to the large sample size, there was adequate statistical power. Some limitations should be acknowledged. Study variables were self-reported and were thus susceptible to bias. Future research could focus on using an objective measurement of PA and ST variables. Furthermore, the cross-sectional design implies that no causal inferences can be made, and future research should use a longitudinal design to analyze the relationship.

## Conclusion

The current findings strongly supported that participation in more PA per week was a positive factor for improved life satisfaction. According to ST, less ST per day was a negative factor for higher life satisfaction. This study also indicated that meeting both guidelines (meeting ST and PA guidelines) was a positive factor for higher life satisfaction. Future studies need to investigate, in large adult samples, PA/ST type and PA/ST’s association with life satisfaction and examine the variation between weekdays and weekends.

## Data availability statement

The original contributions presented in the study are included in the article/supplementary material, further inquiries can be directed to the corresponding author.

## Author contributions

ZC summarized the findings and drafted the manuscript. JS contributed to the formal analysis and editing of the manuscript. ZC and WZ developed the strategy of the manuscript and reviewed and edited the final manuscript. All authors have read and agreed to the published version of the manuscript.
